# Arf GTPase interplay with Rho GTPases in regulation of the actin cytoskeleton

**DOI:** 10.1080/21541248.2017.1329691

**Published:** 2017-11-03

**Authors:** Vikash Singh, Anthony C. Davidson, Peter J. Hume, Daniel Humphreys, Vassilis Koronakis

**Affiliations:** a Department of Pathology, University of Cambridge, Cambridge, UK; b Department of Biomedical Science, University of Sheffield, Sheffield, UK

**Keywords:** actin cytoskeleton, Arf and Rho GTPases, EPEC and bacterial effector proteins, salmonella, wave regulatory complex

## Abstract

The Arf and Rho subfamilies of small GTPases are nucleotide-dependent molecular switches that act as master regulators of vesicular trafficking and the actin cytoskeleton organization. Small GTPases control cell processes with high fidelity by acting through distinct repertoires of binding partners called effectors. While we understand a great deal about how these GTPases act individually, relatively little is known about how they cooperate, especially in the control of effectors. This review highlights how Arf GTPases collaborate with Rac1 to regulate actin cytoskeleton dynamics at the membrane via recruiting and activating the Wave Regulatory Complex (WRC), a Rho effector that underpins lamellipodia formation and macropinocytosis. This provides insight into Arf regulation of the actin cytoskeleton, while putting the spotlight on small GTPase cooperation with emerging evidence of its importance in fundamental cell biology and interactions with pathogenic bacteria.

## Introduction

The actin cytoskeleton comprises a scaffold of polymeric actin filaments that are assembled and disassembled to organize cell architecture and direct many cell processes. One of the key mediators of actin polymerisation is the ubiquitous actin related protein 2/3(Arp2/3) complex, which itself requires activation by nucleation promoting factors (NPFs). Neural Wiskott-Aldrich syndrome protein (N-WASP) and WASP family verprolin homolog (WAVE) are the best characterized of these proteins, and their regulation is of considerable importance.^1^

**Figure 1.**
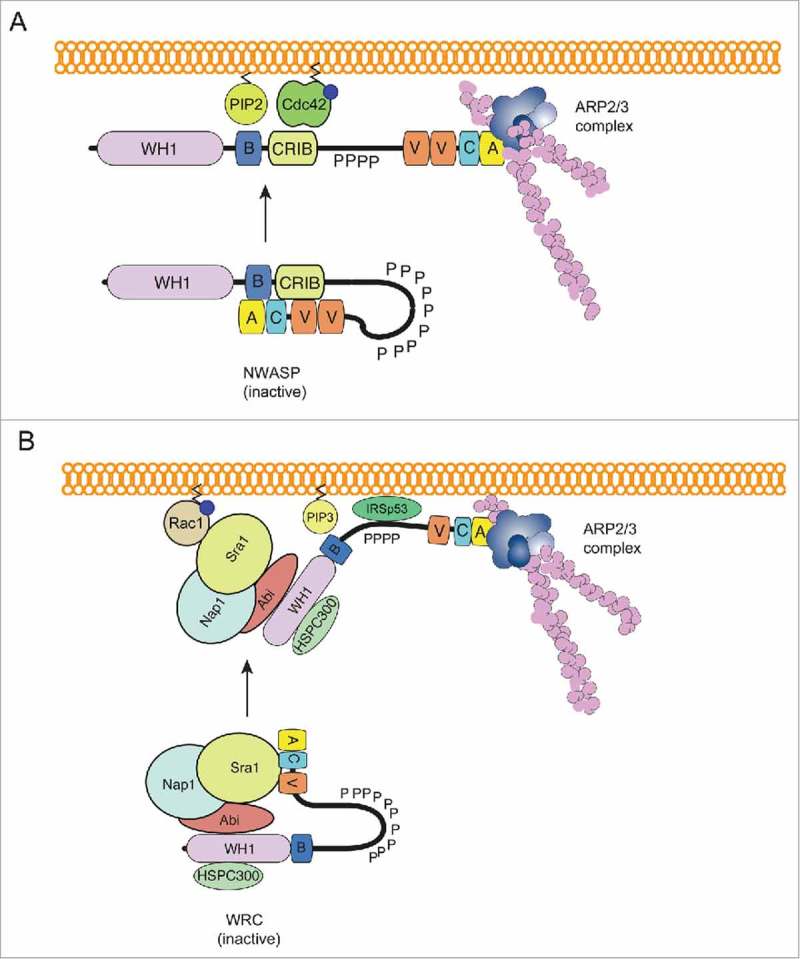
Factors known to regulate NWASP and WRC (A) N-WASP exists in an auto-inhibited state, with its Arp2/3-activating VCA domain bound to the GTPase binding domain (GBD). Binding of PtdIns(4,5)P2 to the polybasic (B) region, and active Cdc42 (GTP bound) to the GBD releases the VCA domain, allowing it to bind the Arp2/3 complex and initiate actin polymerization. (B) Similarly, the VCA domain of WAVE is also concealed, as it is bound to Sra1 within the WRC, rendering the complex inactive. Numerous factors such as binding of GTP-loaded Rac1 to Sra1, binding of PtdIns(3,4,5)P3 to the polybasic (B) domain, and the binding of IRSp53 (and other SH3 containing proteins) to the poly-proline region of WAVE, have been implicated in WRC activation, potentiating actin polymerization.

It is understood that N-WASP exists in an auto-inhibited conformation, with its Arp2/3-activating verprolin homology-cofilin-acidic domain (VCA) shielded by the GTPase binding domain (GBD). Binding of the Rho family GTPase Cdc42 to the GBD releases the VCA domain, enabling it to bind and activate the Arp2/3 complex^2^ as indicated in Fig. 1A. Conversely despite knowing that the Rho GTPase Rac1 can trigger Wave-mediated Arp2/3 activation for over 2 decades, the precise molecular mechanism of regulation remains elusive. WAVE is part of the heteropentameric WRC, comprising of WAVE, Cyfip, Nap1, Abi1 and HSPC300 or their homologues.^3^ Rac1 has been shown to directly interact with Cyfip,^4,5^ however its affinity for the protein is very low (∼10 μM), suggesting that additional factors likely participate in WRC activation. Recent research has identified such factors that may contribute to WRC regulation.^6^ Activation of immunopurified WRC *in vitro* required an electrostatic interaction between the polybasic domain of WAVE and acidic phospholipids such as phosphatidylinositol (3,4,5) triphosphate (PIP_3_), in addition to Rac1 binding^7,8^ as demonstrated in Fig. 1B. Proteins containing SH3 domains such as the IRSp53, Toca1 and WRP interact with proline rich regions of Abi2 and WAVE, and facilitate membrane recruitment and activation of the WRC.^9^ Also many transmembrane receptors such as GPCRs, neuroligins and protocadherins^5^ have been reported to contain a conserved motif termed the WRC-interacting receptor sequence (WIRS) that facilitates the recruitment of WRC to the plasma membrane.^10^ WIRS motifs have been demonstrated to directly interact with a composite surface on Sra and Abi in the WRC, which is unique in that it can only interact with the fully formed complex. Furthermore, phosphorylation of WAVE by proteins such as Abl, Src and Cdk5 kinases is believed to be key players in WRC regulation and may destabilise interactions between the VCA and Sra, promoting activation.^5,11^

Recapitulating WRC activation *in vitro* has uncovered important aspects of its regulation. Recent efforts modeling WRC activation at phospholipid bilayers showed that Rac1 is required, but not sufficient for WRC activation in cell-free extracts. The work found an unexpected requirement for ADP-ribosylation factor^12^ (Arf) GTPases, further implicating involvement of these proteins in cytoskeletal regulation, while opening up the intriguing possibility of 2 GTPases working together to directly modulate a Rho effector.

### Arf driven regulation of actin cytoskeleton dynamics

Arf GTPases are best known for their role in membrane trafficking and vesicle sorting^13^ and, like other GTPases, Arfs act as molecular switches by shuttling between their active GTP-bound and inactive GDP-bound conformations. Hydrolysis of bound GTP is stimulated by GTPase-activating proteins (GAPs), whereas the exchange of GDP to GTP is mediated by guanine nucleotide exchange factors (GEFs). The 6 mammalian Arfs are grouped into 3 classes on the basis of sequence homology, class I (Arfs 1–3), class II (Arfs 4–5) and class III (Arf6). While the class I and II Arfs are primarily localized in and around the Golgi apparatus,^14,15^ Arf6 is found predominantly at the plasma membrane and on a subset of endosomes.^16^

The involvement of Arfs in actin dynamics has primarily been attributed to their ability to activate lipid-modifying enzymes, which alter the membrane microenvironment. Arfs are capable of directly modulating local phosphoinositide synthesis, which has an impact on various actin regulatory proteins. Arfs have also been implicated in indirect activation of Rho GTPases. For example, active Arf6 recruits the bi-partite Rac GEF Dock 180-ELMO,^17^ likely due to local PI(4,5)P2 generation at on the plasma membrane at the leading edge of a cell, stimulating Rac activation.

Arf6 may also modulate Rac activity by controlling the availability of lipid raft components,^18^ due to its role in endosomal recycling, which has been shown to be instrumental in the attachment and spreading of anchorage-dependent cells. Furthermore, inhibiting the activity of Arfs has been shown to directly impact Rac-dependent membrane ruffling,^19^ phagocytosis^20^ and breast cancer cell migration.^21^

Arf proteins have also been shown to down-regulate the activity of Rho GTPases. At Golgi membranes, Arf1 down-regulates Cdc42 activity by recruiting ARHGAP21,^22,23^ a cdc42 GAP. Another interesting aspect, which further enforces the notion that Arf GTPases coordinate regulation of actin cytoskeleton, is the interaction between Arf GAPs and Rho GEFs. The best-known example of this unique mode of actin remodeling is the interaction between Arf GAP GITI, and β-Pix, a Rac GEF. GIT1 forms a complex with β-Pix, and inhibits the activity of Rac1 at the leading edge of cells.^24,25^

### Arf regulation of the WAVE Regulatory Complex

Despite the plethora of research implicating Arf GTPases in cytoskeletal remodelling there has been little evidence for their direct interaction with actin regulators such as NPFs. Arf1 has though been implicated in the recruitment of both Rac1 and the WRC component CYFIP to the trans-Golgi network^26^ (TGN). Here it aids in the generation of AP1-Clathrin coats, also promoting membrane tubulation as a result of NWASP driven Arp2/3 complex dependent actin polymerisation. The precise role of Arf in regulating full WRC activation at the plasma membrane though has not been outlined.

Reconstitution studies in cell-free extracts showed that both PI(4,5)P2 and PI(3,4,5)P3 recruited the WRC and Rac, yet remarkably the WRC was only activated on PI(3,4,5)P3^12^. The difference was found to be the activation status of Arf GTPases, which although present on both lipids, were only GTP-bound (active) on PI(3,4,5)P3. *In vitro* binding studies with purified components showed that Rac1 and Arf1 were individually able to bind weakly to recombinant WRC and poorly activate it, but when both GTPases were anchored at the membrane, recruitment and concomitant activation of WRC were dramatically enhanced. This cooperativity between the 2 GTPases was sufficient to polymerize actin filaments in a WRC-dependent manner that propelled phospholipid-coated beads through cell-free extracts.

**Figure 2.**
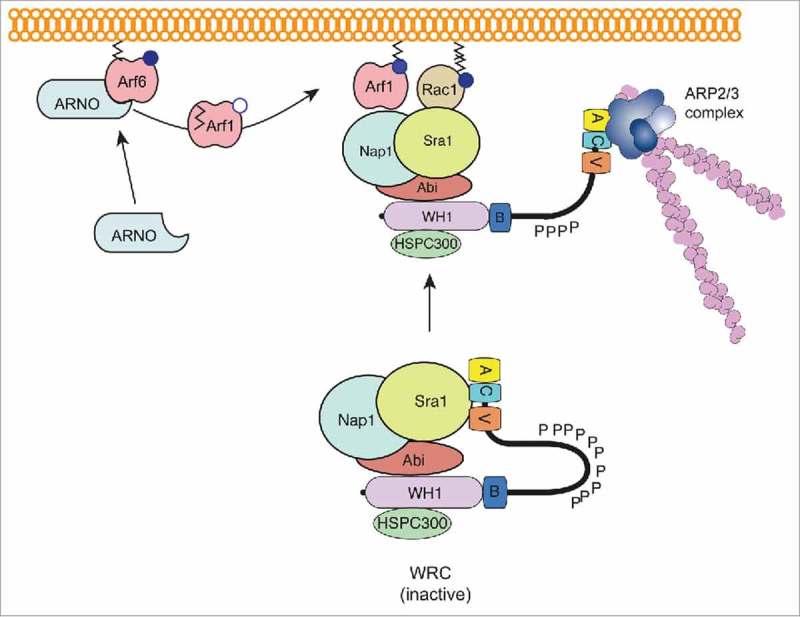
Collaboration between Arf and Rho GTPases to potentiate actin assembly via WRC. The Wave regulatory complex (WRC) exists in an inactive state i.e. the VCA domain of WAVE is not free to bind to the Arp2/3 complex to induce actin polymerization. Upon external stimuli, such as effector protein delivery by *Salmonella* or on EGF stimulation, Arf6 recruits and activates ARNO that in turn stimulates the exchange of GDP (white circle) bound to Arf1 for GTP (blue circle). Activated Arf1 consequently anchors via its exposed myristoylation moiety (black lines) to the plasma membrane. The Arf1 binding partner remains unclear, but nevertheless membrane-anchored active Arf1 and Rac1 work in cooperation to recruit and activate the WRC (i.e., release the VCA domain) that induces Arp2/3-dependent polymerization of actin filaments (pink).

The recruitment and activation of WRC at the membrane is not restricted to Arf1, as the related Arf5, and Arl1, a distant member of the Arf GTPase family, could also achieve similar activity. These key findings suggest that the Arf GTPase family have overlapping or partially redundant functions. Arf6, which is predominantly found to be associated with the plasma membrane, was also found to regulate actin assembly via the WRC.^27^ Unlike, other Arf family members, Arf6-mediated actin polymerization was not achieved by direct interaction with the WRC but instead through recruitment of the Arf GEF ARNO, which acts at the plasma membrane where it recruits and activates Arf1 to collaborate with Rac1. This highlights the spatiotemporal coordination between 2 distinct classes of small GTPase that underlies actin polymerization at the plasma membrane, as described in Fig. 2. This biochemical work has been reinforced with evidence demonstrating that Arf plays an important role in WRC regulation in the cell.

Identifying phenotypic changes in actin dynamics in mammalian cells is problematic due to redundancy of many actin regulatory proteins, especially true for the Arf GTPases. Fortunately *Drosophila melanogaster* has only one member in each of the Arf classes. *Drosophila* S2R+ cells form characteristic lamellipodia with the cells appearing uniformly round when adherent. Depletion of any individual component of the WRC, or Rac1 has been demonstrated to abolish lamellipodia formation. Depletion of the Arf1 homolog Arf79f^28^ in S2R+ cells also abolished lamellipodia formation with cells appearing spiky, characteristic of loss of a WRC dependent activity. Interestingly, the expression of human Arf1 resulted in restoration of lamellipodia in Arf79 depleted *Drosophila* cells. However, the expression of active Rac1 in these cells failed to restore lamellipodia formation, further signifying the direct importance of Arf. Consistent with this, active Arf79F was critical for Sra1 localization and concomitant generation of lamellipodia both in cells as well as *in vitro*.^28^ Furthermore, a recent study demonstrated that Arf6 potentiated the formation of Rac1 and Wave dependent ventral F actin rosettes in breast cancer cells upon epidermal growth factor (EGF) stimulation.^29^ In addition, the authors could demonstrate that interference with ARF6 expression resulted in poor activation and plasma membrane localization of Rac1 in response to EGF treatment. The study highlights a potential role for ARF6 in linking EGF-receptor signaling to Rac1 recruitment and activation at the plasma membrane to promote breast cancer cell directed migration.

### *Salmonella* manipulates Arf GTPases to activate the WAVE Regulatory Complex

Bacterial pathogens manipulate the cytoskeleton^30^ to establish infections and have long been used to better understand how actin dynamics are being regulated including those governing the WRC. *Salmonella enterica* (hereafter *Salmonella*) is a Gram-negative facultative intracellular pathogen that infects and colonizes vertebrate hosts with outcomes ranging from sub-clinical infections to life-threatening systemic disease. Upon contact with a host cell, *Salmonella* translocates a cohort of virulence effector proteins into the host cell via its Type III Secretion System.^31^ Some of these effector proteins enter the cytosol where they are able to remodel the actin cytoskeleton resulting in host membrane ruffling that drives *Salmonella* entry via macropinocytosis.^32^ It is known that *Salmonella* requires the WRC to generate membrane ruffles,^32^ which is mediated by targeting small GTPase signaling pathways. The virulence effectors SopE and SopE2 mimic host cell GEFs^33,34^ by triggering the activation of Rac1 and Cdc42, and Cdc42 alone respectively. *Salmonella* utilizes SopE to recruit WRC in a Rac1 dependent manner.^35^ However as already indicated Rac1 alone is not sufficient to activate WRC,^12^ and would need an activated Arf to drive WRC dependent actin assembly. *Salmonella* does not encode any known Arf GEF, therefore to activate Arf and subsequently the WRC, the pathogen targets the network of host Arf GEFs.^36^

*Salmonella* targets and recruits the host GEF ARNO^35^ (also known as cytohesin 2) to pathogen entry foci to activate Arf1, which cooperates with SopE-activated Rac1 to drive WRC dependent actin assembly.^27^ ARNO is maintained in the cytosol in an auto-inhibited conformation, but is recruited and activated at the plasma membrane via Arf6 and acidic phospholipids such as PI(3,4,5)P3.^37^ Interestingly there are 2 splice variants of ARNO that differentially interact with phospholipids, the presence of 3 glycine residues within the PH domain (3G), results in recruitment to PI(4,5)P2, whereas a double glycine version (2G), is preferentially recruited to PI(3,4,5)P3.^38^ It is highly probable that these different variants have distinct biological functions, with only the 2G variant being shown to promote the production of Rac1 dependent ventral actin structures in Beas-2b and HeLa cells upon Phorbol myristate acetate (PMA) stimulation.^39^ With Arf and PI(3,4,5)P3 already identified as being important for WRC driven actin assembly, this result is not surprising.

The recruitment of ARNO to the membrane by Arf6 triggers WRC-dependent actin polymerization and *Salmonella* uptake via Arf1^27^. ARNO recruitment to invasion sites is also aided by host Arf6 GEFs EFA6 and BRAG2 as well as PI(3,4,5)P3 production via the *Salmonella* effector SopB. Surprisingly, efficient *Salmonella* entry also requires host Arf GAPs, inactivators of Arf signaling.^40^ This suggests that cycles of GTPase activation and inactivation facilitate the actin polymerisation required for pathogen uptake. *Salmonella* thus exploits a remarkable interplay between both host- and bacteria-derived GEFs and GAPs to subvert the cytoskeleton and force entry into non-phagocytic cells.

### *Escherichia coli* interfere with Arf signaling to block WAVE Regulatory Complex activation

Enteropathogenic and enterohemorrhagic *E. Coli* (EPEC and EHEC) are major global threats to human health that cause acute gastroenteritis and bloody diarrhea respectively.^41^ Unlike *Salmonella*, EPEC and EHEC are extracellular pathogens. They use their T3SS to secrete numerous virulence effector proteins targeting the actin cytoskeleton to form cell surface pseudopodia called actin pedestals where they establish infection.^42^ Actin pedestals enable both EPEC and EHEC to colonise the surface of intestinal epithelial cells, resulting in distinctive ‘lesions’ characterized by the destruction of brush border microvilli characteristic to these cells. As a result the pathogen is able to escape into the basolateral region, where the bacteria encounter macrophages. Both EPEC and EHEC use multiple mechanisms to avoid being engulfed by the infiltrating professional phagocytes.^43^ Macrophages facilitate uptake of foreign bodies through a process of actin driven phagocytosis.^44^ Phagocytosis in part is driven via WRC dependent actin assembly, a process that requires cooperating Arf and Rac1 GTPases.^45^ In an attempt to evade this process EPEC likely interferes with one or more of these components. The effector protein EspG, know to interact with Arf1 was thus an intriguing candidate to investigate. EspG is conserved across EPEC, EHEC and *Citrobacter*, and was originally described as a homolog of VirA in *Shigella*.^46^ EspG has multiple known functions, and acts as a molecular scaffold by simultaneously binding p21-activated kinases (PAK) and GTP bound Arf GTPases.^47^ EspG is also known to act as a Rab-GAP and interferes with Golgi signaling.^47,48^ EspG was found to incapacitate WRC activation via a dual mechanism.^45^ Firstly, EspG binding to Arf1 impedes cooperation with Rac1, thereby inhibiting WRC recruitment and activation. Further investigation of the mechanism by which EspG incapacitates WRC, identified key residues in the Arf1 α−1 helix and switch-1 domain, which might be critical for WRC activation. In addition EspG's interaction with Arf6 sterically hinders its interaction with Arno,^45^ thereby preventing Arf1 activation and consequent WRC-mediated phagocytosis. Another EPEC/ EHEC injected effector protein EspH has been previously shown to inhibit actin driven phagocytosis by disrupting the actin cytoskeleton. EspH inactivates host Rho GTPases^49^ such as Rac1 by directly binding to Rho GEFs that are needed for activation of Rho GTPases.

Thus, manipulation of the WRC underpins diverse virulence strategies where invasive intracellular pathogens activate the WRC while extracellular pathogens inhibit the WRC.

## Conclusion

It is well established that Arf GTPases are involved in vesicle trafficking and they have long been implicated in regulating the actin cytoskeleton. However, until now there has been scant evidence for direct regulation of NPFs. Arf GTPases, in particular Arf1, coordinate with Rac to activate WRC and facilitate lamellipodia formation. The ability of pathogens to target Arf GTPases that manipulate the host actin cytoskeleton to establish infection, further strengthens the significance and prime importance of these small GTPases in regulating critical processes at the plasma membrane.

## Future prospective

Despite linking and underlining the importance of Arf GTPases in WRC regulation, the precise means by which this is achieved is still uncertain. A possible interaction between Arf1 and the WRC component Nap1 has been reported.^12^ Even so, to date there is no conclusive evidence of a direct interaction between Arf and any component of the WRC, with an identified binding site still elusive. It is possible that the regulation of WRC by Arf GTPases is not dependent on any direct interactions watsoever. Indeed manipulation of the local environment, or more interestingly other key players, such as Rac1 may be the most important function of Arf here. Cooperation between GTPases is becoming of increasing interest, but understanding how this is achieved is challenging. Whether Arf directly modulates Rac to potentiate its affinity for the WRC, or physically blocks or recruits other proteins that are involved in activation of the complex is something that needs to be investigated.

Pathogens, as discussed here are great tools to enhance our understanding of basic cell biology. They continue to prove invaluable assets in the biologist's quest to better comprehend not only Arf-Rac cooperativity, but also the potential interaction and cooperation of other small GTPases, and the actin cytoskeleton. Multiple pathogens have evolved to intricately manipulate host cells in the most efficient manner, and as with *Salmonella* and the WRC, they likely hijack numerous as yet unidentified fundamental pathways.
